# Regionally Discrete Aqueous Humor Outflow Quantification Using Fluorescein Canalograms

**DOI:** 10.1371/journal.pone.0151754

**Published:** 2016-03-21

**Authors:** Ralitsa T. Loewen, Eric N. Brown, Pritha Roy, Joel S. Schuman, Ian A. Sigal, Nils A. Loewen

**Affiliations:** 1 Department of Ophthalmology, School of Medicine, University of Pittsburgh, Pittsburgh, United States of America; 2 Department of Ophthalmology, School of Medicine, Vanderbilt University, Nashville, United States of America; 3 The Louis J. Fox Center for Vision Restoration, School of Medicine, University of Pittsburgh, Pittsburgh, United States of America; Duke University, UNITED STATES

## Abstract

**Purpose:**

To visualize and quantify conventional outflow directly in its anatomic location.

**Methods:**

We obtained fluorescein canalograms in six porcine whole eyes and six porcine anterior segment cultures. Eyes were perfused with a constant pressure of 15 mmHg using media containing 0.017 mg/ml fluorescein. Flow patterns were visualized using a stereo dissecting microscope equipped for fluorescent imaging. Images were captured every 30 seconds for 20 minutes for time lapse analysis. Anterior chamber cultures were imaged again on day three of culture. Canalograms were first analyzed for filling time per quadrant. We then wrote a program to automatically compute focal flow fits for each macropixel and to detect convergent perilimbal flow patterns with macropixels grouped into 3 equal-radial width rings around the cornea. A generalized additive model was used to determine fluorescence changes of individual macropixels.

**Results:**

The resulting imaging algorithm deployed 1024 macropixels that were fit to determine maximum intensity and time to fill. These individual fits highlighted the focal flow function. In whole eyes, significantly faster flow was seen in the inferonasal (IN) and superonasal (SN) quadrants compared to the superotemporal (ST) and inferotemporal (IT) ones (p<0.05). In anterior chamber cultures, reduced flow on day 1 increased in all quadrants on day 3 except in IT (p<0.05). Perilimbal ring analysis uncovered convergent perilimbal flow.

**Conclusions:**

An algorithm was developed that analyzes regional and circumferential outflow patterns. This algorithm found flow patterns that changed over time and differ in whole eyes and anterior segment cultures.

## Introduction

Although bulk outflow of aqueous humor can be estimated with fluorophotometry[1] and tonography,[2] no method exists to quantify segmental outflow. Dye[3,4] and cast preparation[5] had been used more than a century and a half and a century ago, respectively, to better understand the outflow tract anatomy. Benedikt[6] provided a comprehensive case series of fluorescein filling patterns in 30 glaucoma patients who received an intracameral bolus at the slit lamp. More recently, spectral domain optical coherence tomography (SD-OCT) has been used to describe the aqueous spaces of the conventional outflow pathway in human eyes.[7] The ability to measure fluid movements in small caliber vessels in general is limited and often relies on the detectability of moving particles such as blood cells (e.g. when measuring flow in retinal arteries by SD-OCT).[8] In order to visualize the slower moving aqueous humor that does not contain signal-reflecting particles, gold nanorods[9] can be utilized, although they may also be enveloped by phagocytic trabecular meshwork (TM) cells and cause in vivo toxicity.[10]

The absence of an automated, standardized method to quantify aqueous humor outflow at a discrete anatomic location, analogous to quantitative coronary angiography,[11,12] has been an obstacle in glaucoma research. Such a technique would be especially useful for investigations into outflow resistance elements that may be downstream of the trabecular meshwork implicated in glaucoma,[13] and have been suggested to be the cause for late failure of canal-based microincisional glaucoma surgeries.[14] It would also allow to investigate the hypothesized focal outflow enhancement of early cell[15] and gene therapy[16,17] based strategies.

We hypothesized that a method of regionally discrete outflow measurements could be developed, termed here quantitative canalography, to detect differences between the ex vivo eye culture system (anterior chamber perfusion cultures) and whole eyes. Commonly used in glaucoma research,[18] the anterior chamber culture model involves a ring that compresses the sclera along the equator to secure the anterior segment, which may impede flow through episcleral veins. We used standard fluorescence visualization equipment and open source statistical software to develop image processing algorithms that capture focal flow patterns of fluorescein.

## Methods

### Eye Cultures

We obtained freshly enucleated porcine eyes from a local abattoir (Thoma Meat Market, Saxonburg, PA) and cultured them within 2 hours. Eyes were placed into ophthalmic 5% povidone-iodine (Betadine, Alcon, Fort Worth, TX) for 3 minutes, then rinsed with and kept in phosphate buffered saline (PBS, Gibco,Thermo Fisher Scientific, Waltham, MA). We determined whether an eye was left or right and marked the medial and superior peripheral cornea for orientation and mounting. Perfused anterior chamber cultures[18] were compared to perfused whole eyes.

#### Whole eye perfusion

For the whole eye perfusion studies, adnexal structures, consisting of lids, nictitating membrane, orbital fat, were removed and the conjunctiva and muscles were trimmed up to the level of the posterior pole. The eye was then placed with the optic nerve into the cap of a plastic tube for a compression-free mount (CryoElite Cryogenic Vial #W985100, Wheaton Science Products, Millville, NJ). Six eyes were perfused with phenol red free Dulbecco's modified Eagle's media (DMEM) containing 100 units/mL of penicillin, 100 μg/mL of streptomycin, and 0.25 μg/mL of amphotericin B (Antibiotic-Antimycotic (100X), Gibco, Thermo Fisher Scientific, Waltham, MA) at a constant pressure of 15 mmHg. We cannulated the anterior chamber with a 30 gauge needle on a 3 way stopcock that was connected via polyethylene tubing to a 20 ml syringe without the plunger, which acted as a reservoir. The needle was always inserted nasally with the needle bevel up and precisely centered in the anterior chamber. The top of the fluid column in the syringe was held at a height of 20 centimeters above the center of the anterior chamber. The resulting intraocular pressure of 15 mmHg was confirmed with a pressure transducer (DTX-plus, Argon Medical Devices, Plano, TX; amplifier, ADInstruments, Colorado Springs, CO, USA) that was connected with a three way connector at the the same level. The intracameral needle was inserted just anterior to the limbus and advanced into the center of the pupil.

#### Anterior segment culture perfusion

Anterior chamber cultures were prepared by removing adnexal structures and muscles to the level of the equator using 0.5 mm toothed forceps and Steven’s scissors. A full thickness scleral incision was fashioned with a type 15 scalpel and the eye was hemisected along the equator with a circumferential cut using Steven’s scissors. The anterior half was grasped with serrated forceps and the choroid was separated from the sclera. The entire uvea including ciliary body and iris was gently detached and peeled away in one piece with the lens still attached. Anterior segments were repeatedly rinsed with the same media as as used for perfusion. Anterior segments were mounted in perfusion dishes as described before[19,20] using a standard scleral compression ring. The eyes were first pressurized with DMEM at 15 mmHg before being switched to DMEM containing fluorescein as described below.

### Fluorescent Imaging

**Fig 1:**
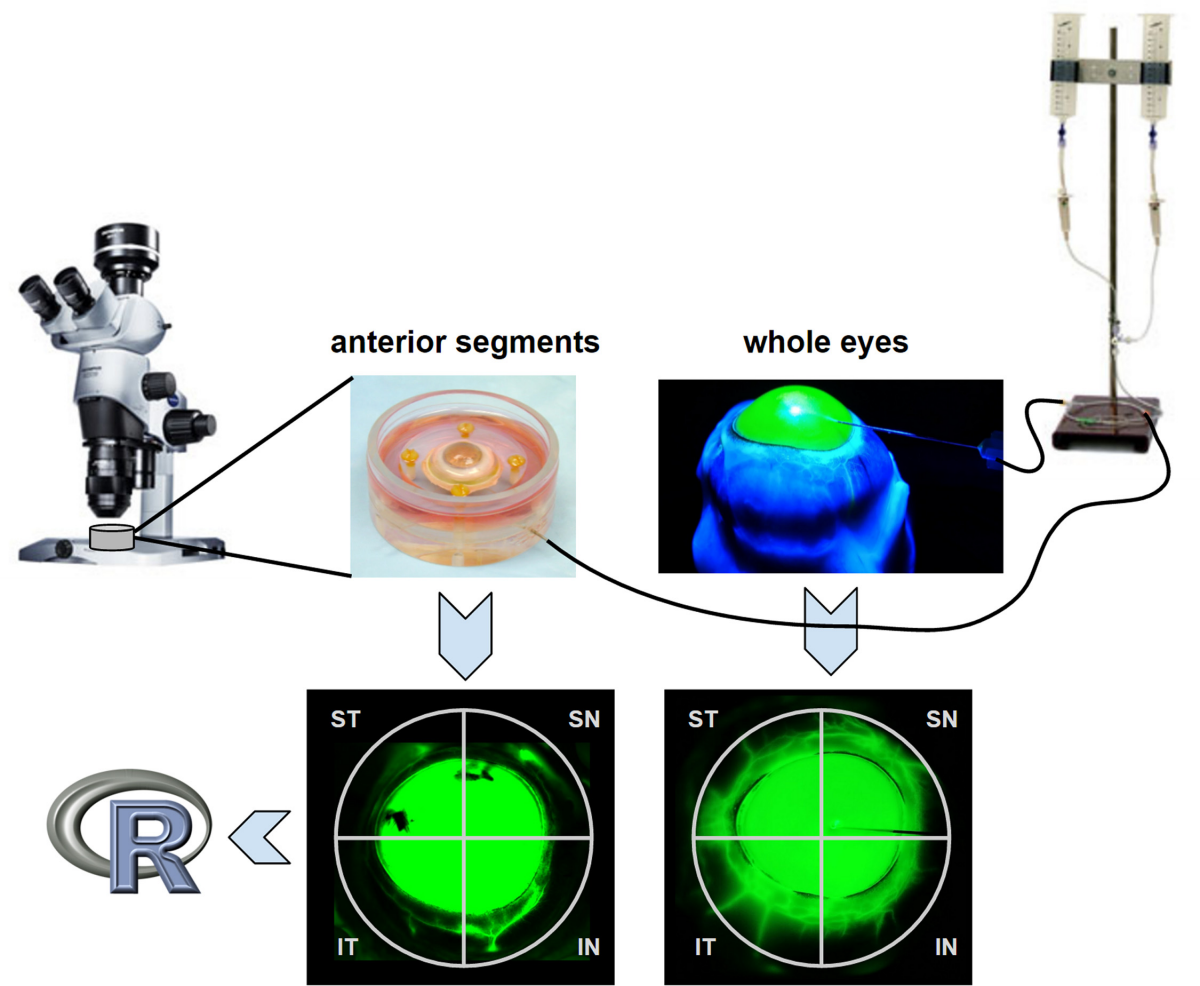
Imaging equipment and gravity perfusion of anterior segment culture (left) and whole eye mounts (right). Frontal view canalograms (bottom). Image data was processed using open source software environment, R. IN = inferonasal, SN = superonasal, ST = superotemporal, IT = inferotemporal quadrant. R = free software programming environment for statistical computing and graphics.

The perfusion of whole eyes and anterior chamber cultures was switched to DMEM containing 0.017 mg/ml fluorescein at 15 mmHg after a fluid exchange to fill the entire chamber with the same. Chromophore flow patterns were visualized (Fig 1) using a stereo dissecting microscope equipped for fluorescent imaging (Olympus SZX16 with GFP filter cube and DP80 Monochrome and Color Camera; Olympus Corp., Center Valley, PA). Images were acquired every 30 seconds for 20 minutes for time lapse analysis (CellSens, Olympus Life Science, Tokyo, Japan) with a resolution of the binned image capture of 580 by 610. Anterior chamber cultures were imaged again on day 3 of culture in the same fashion. Whole eyes could not be maintained for the same period of time.

### Image Analysis

The images were divided into the inferonasal (IN), superonasal (SN), superotemporal (ST) and inferotemporal (IT) quadrants. Filling time was calculated as a difference between the beginning of the time lapse and first presentation of fluorescence in the aqueous vessels.

### Regional Outflow and Perilimbal Aqueous Flow

**Fig 2:**
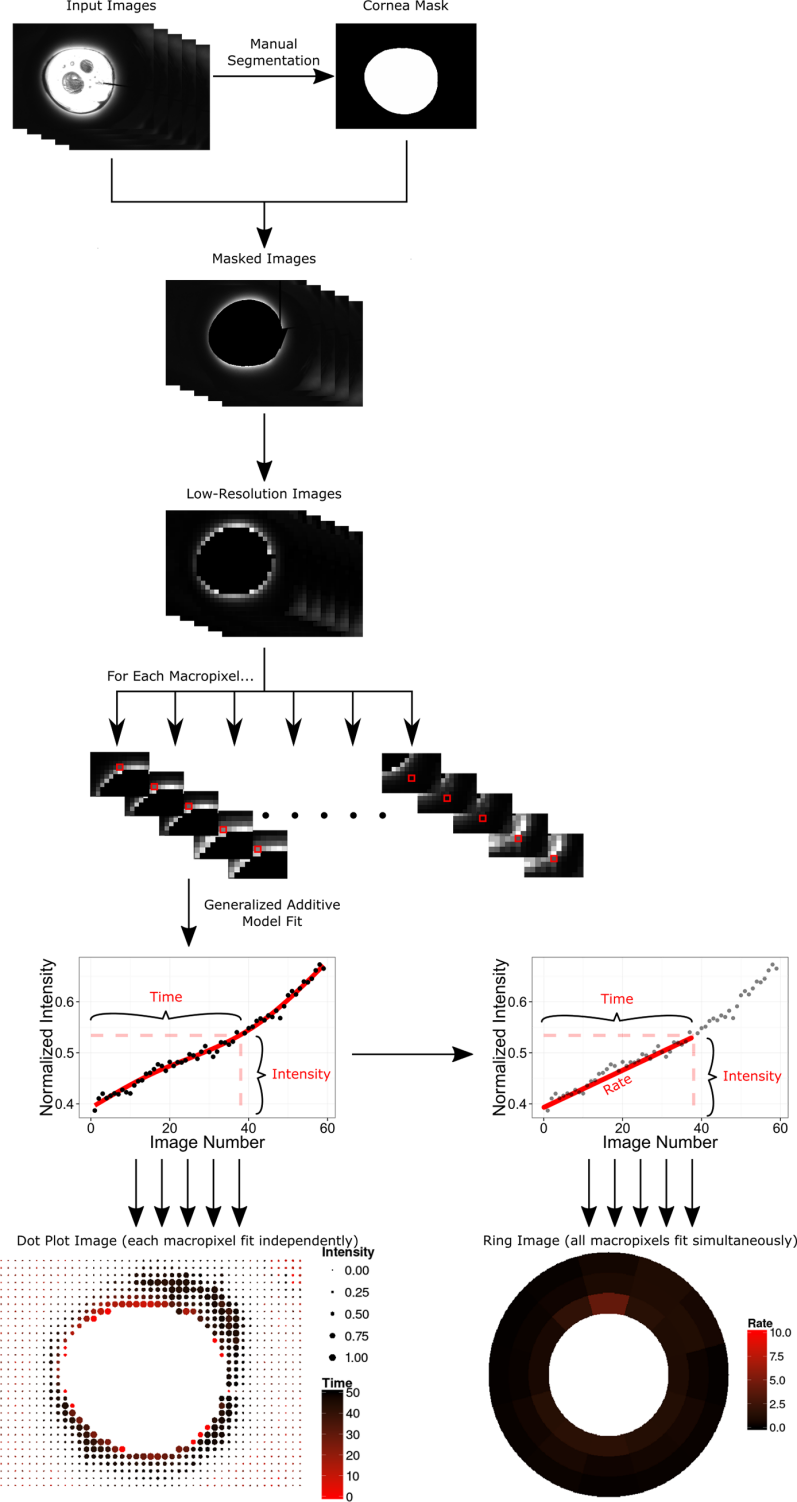
Image processing pipeline performed by the eye-canalogram R statistical analysis package. The left lower part of the figure shows how individual fits were generated to create a dot plot image–a single model fit was used for each macropixel. The lower right figure shows how filling rate was computed to create ring plots in which one global model fit was used for the entire plot.

To test our hypothesis of outflow being altered in anterior chamber cultures, we created a mathematical model in the R Programming Environment for Data Analysis and Graphics.[21] This program computed the focal outflow as well as the convergent perilimbal aqueous flow based on image data using the EBImage[22] and mgcv packages.[23] The code has been made available in its entirety at https://github.com/enbrown/eye-canalogram/. In short, this algorithm loaded all images from one canalogram and reduced their resolution to 32 by 32 macropixels (Fig 2). The cornea and compression ring of anterior segment cultures were individually identified for each canalogram and were excluded from subsequent processing. Only pixels outside of the cornea and forming a complete ring around the eye were used. Each macropixel in the reduced resolution image was fit individually to a generalized additive model (GAM). The half-maximum fluorescence intensity and time in seconds to reach this intensity were computed from the GAM fit and plotted on a dot plot. The size of the pixels corresponded to the intensity of fluorescence (the more intense the fluorescence signal, the larger the macropixel), while the color of the pixels corresponded to the rate of filling (the faster the filling the more red the macropixel).

In addition, macropixels were grouped by clock hour and into 3 equal-width perilimbal rings surrounding the cornea creating 36 regions of interest. A single generalized additive model was fit to model all 36 regions using smoothing terms for the clock hour, radial ring, and frame number. From this fit, the time to reach half-maximum fluorescence and the rate of filling were computed for each region and displayed in a ring plot. Filling rate was defined as the increase in absolute fluorescence from an initial value of zero to the fluorescence intensity at half-max divided by the time taken to reach half-max intensity.

The increase in fluorescence from the initial fluorescence in the first frame to the time at half-maximal fluorescence was determined for each macropixel individually based on the global fit in units of “percent fluorescence per frame” and then summed to give the total flow. The proportion of the total flow for the entire analysis region excluding the cornea or anterior segment compression ring was then attributed to each quadrant and converted to units of microliters per minute using the established flow of 3 microliters per minute (see example in S1 Fig).[24]

## Results

**Fig 3:**
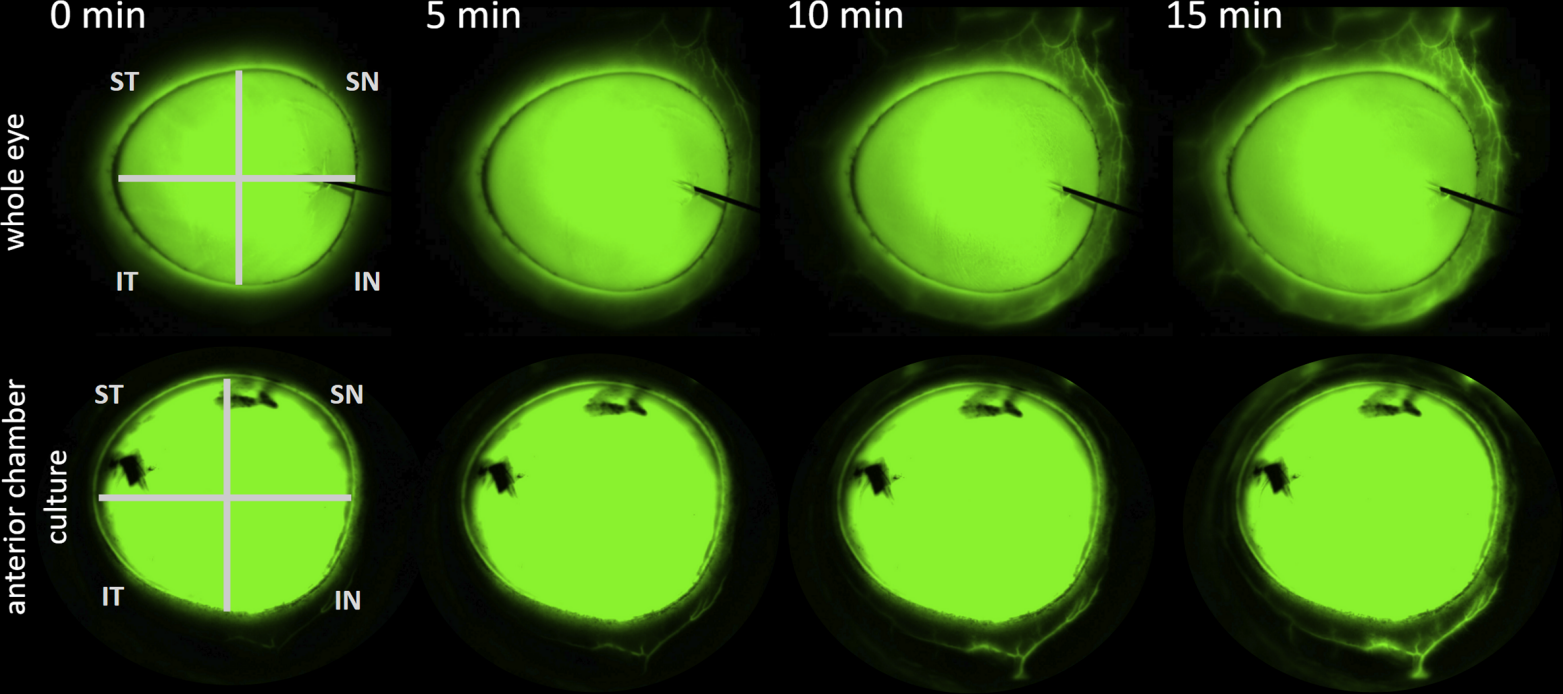
Example canalogram time lapse of a whole eye (top) and an anterior chamber culture (bottom). Time is indicated in minutes at the top. IN = inferonasal, SN = superonasal, ST = superotemporal, IT = inferotemporal quadrant. Dye marks which were used during pilot experiments can be seen on the cornea blocking fluorescence.

Imaging with a standard dissection microscope with eGFP/FITC filter allowed visualization of the aqueous route up to the equator in whole eyes and up to the compression ring in anterior chamber cultures. Whole eyes had major drainage structures in all 4 quadrants and showed early outflow in the nasal quadrants (Fig 1 right and Fig 3 top). Aqueous outflow paths could be distinguished from venous and arterial vessels that did not fill. Intricate vascular tree filling patterns could be observed down to 20 micrometer diameter. Flow was present over the entire length of the conjunctiva in whole eyes. In contrast, ASC cultures had a less diffuse drainage pattern with a more focal filling pattern in one or two quadrants and little flow in the remainder (Fig 3, bottom).

**Fig 4:**
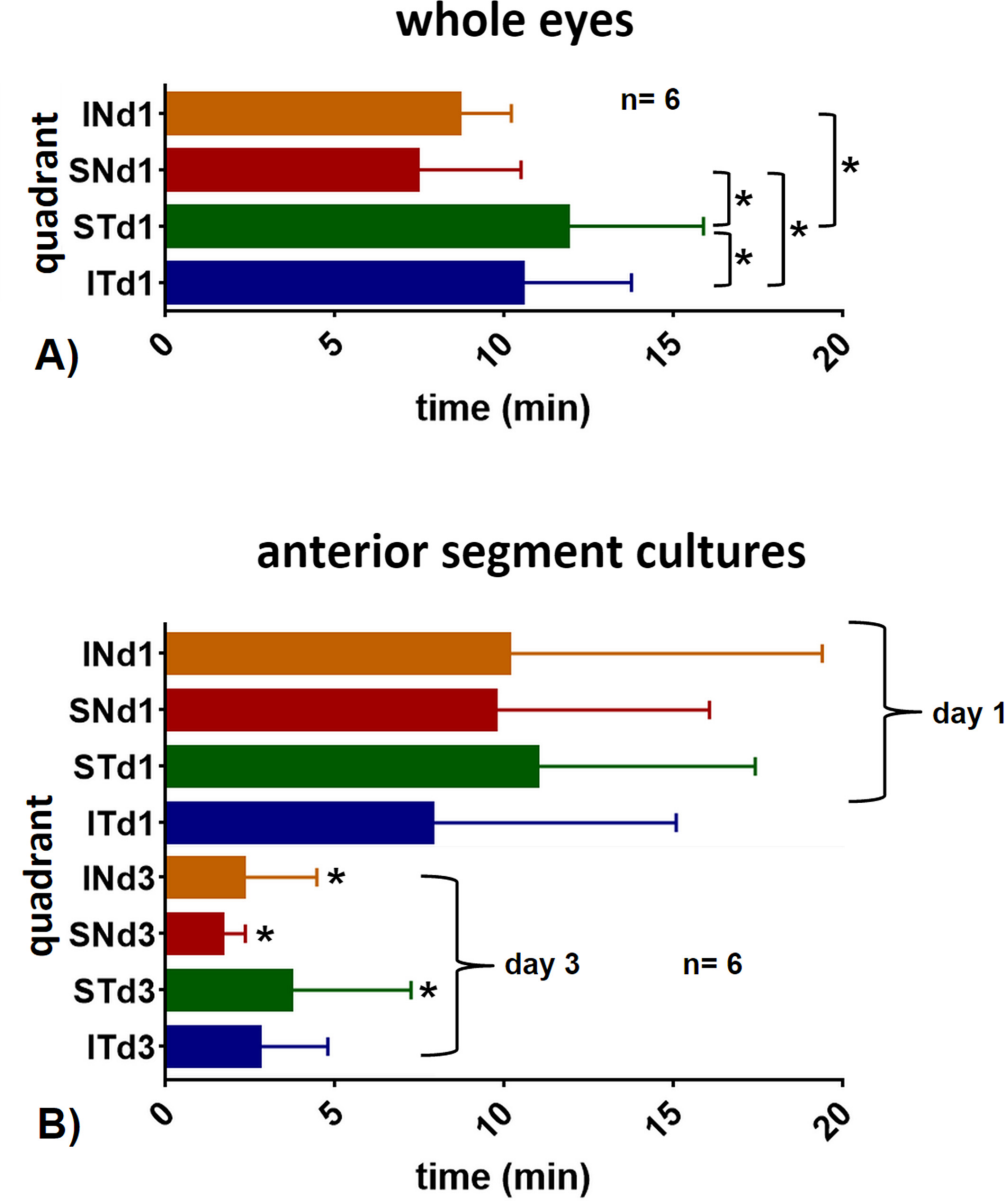
Average outflow time per quadrant. A) In whole eyes, filling was faster nasally (IN, SN) than temporally (ST, IT; *p<0.05, paired t-test). B) Anterior segment cultures (bottom) had a filling time that was slightly slower on day 1 (d1) compared to whole eyes (top). Day 1 filling times of anterior chamber cultures were slower than on day 3 (d3; comparison of same quadrants on day 1 to day 3; *p<0.05, paired t-test). IN = inferonasal, SN = superonasal, ST = superotemporal, IT = inferotemporal quadrant.

In whole eyes, significantly faster flow was seen in the inferonasal (IN) and superonasal (SN) quadrants compared to the superotemporal (ST) and inferotemporal (IT) ones (Fig 4A, p<0.05). There was no statistically significant difference or quadrant preference observed in anterior chamber cultures (Fig 4B, p>0.05). In anterior chamber cultures, reduced flow on day 1 increased in all quadrants on day 3 except in IT (p<0.05). Whole eyes had an imputed flow of 0.9±0.4 microliters per minute (μl/min) in the inferonasal quadrant (IN), of 1.1±0.2 μl/min in the superonasal quadrant (SN), 0.6±0.2 μl/min in the superotemporal quadrant (ST) and 0.4±0.3 μl/min in the inferotemporal quadrant (IT). Anterior chamber cultures on day 1 had a flow of 0.8±0.1 μl/min in the IN, 0.5±0.4 μl/min in the SN, 0.4±0.1 μl/min ST and 0.5±0.2 μl/min in the IT quadrant. Anterior chamber cultures on day 3 had a flow of 2.6±0.3 μl/min in the IN, 2.7±0.4 μl/min in the SN, 1.4±0.2 μl/min ST and 1.9±0.1 μl/min in the IT quadrant. Whole eyes had an average filling time of 7.4 minutes compared to 9.7 minutes in anterior chamber cultures. On day 3, anterior chamber cultures had a faster outflow with a pattern that was closer to whole eyes with preferential nasal drainage, similar to whole eyes (Fig 4). Whole eyes could not be reliably cultured for 3 days due to high metabolic requirements by the posterior segment.

**Fig 5:**
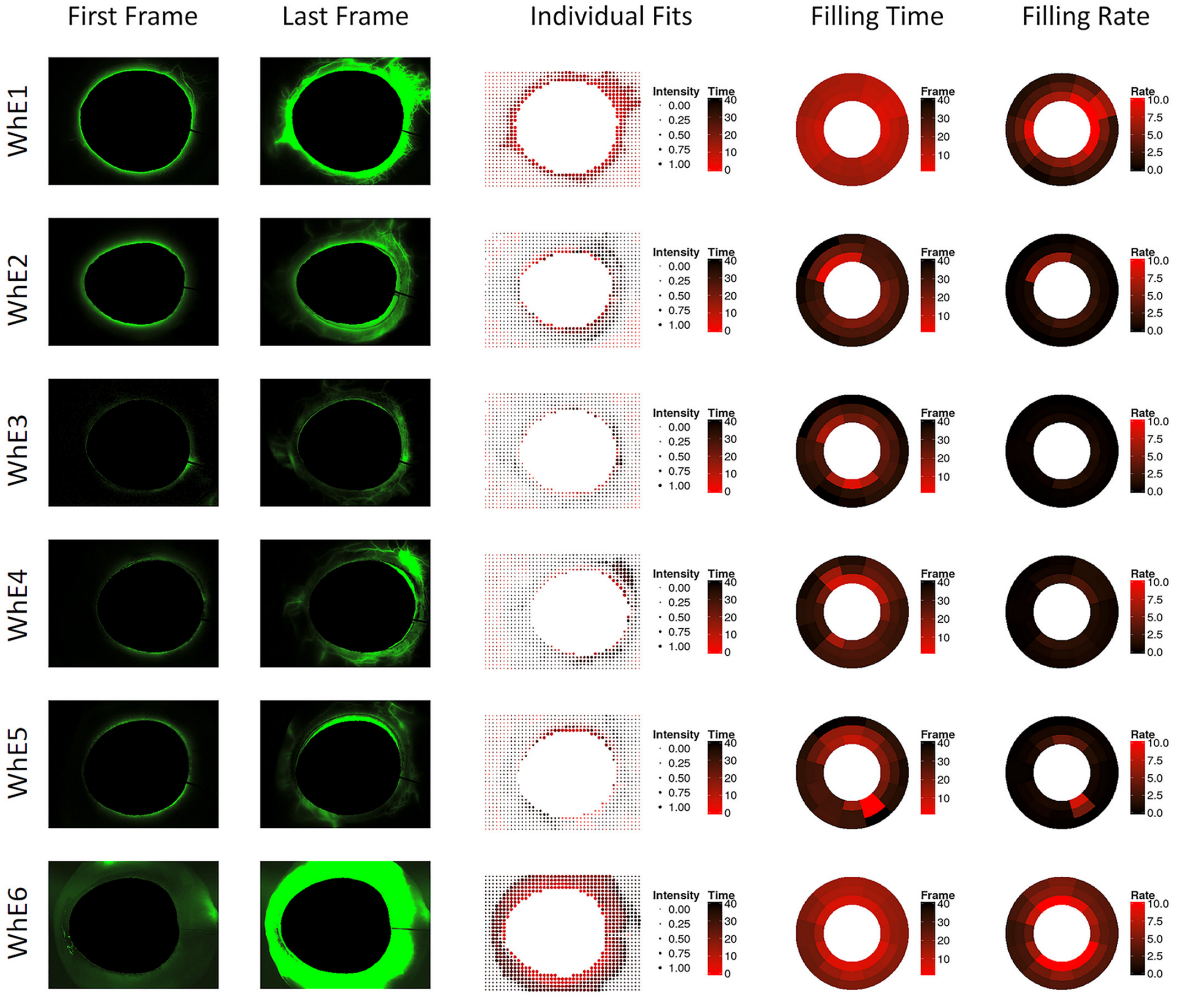
Whole eye (WhE 1–6) canalograms. Shown are images of the first and final image analyzed (left), quantitative canalography dot plots using individual fits (center), and ring plots showing modeled perilimbal aqueous filling time and filling rate (right). WhE = whole eyes, numbered consecutively. All images are rotated to fit the quadrants pattern used in the other figures (SN, IN, IT, ST in clockwise order).

The dot plot with individual fits captured the dynamic filling of drainage vessels well (Fig 5). Red, large dots, indicating early, intense filling, matched the pattern of large aqueous vessels but not that of smaller ones with less intense filling. The pattern of faster nasal filling observed in the manual analysis was also seen in the individual quantitative canalogram of using dot plots. In whole eyes, the analysis of perilimbal flow detected a circular, limbus parallel pattern that spanned several clock hours before converging into larger aqueous vessels (Fig 5).

**Fig 6:**
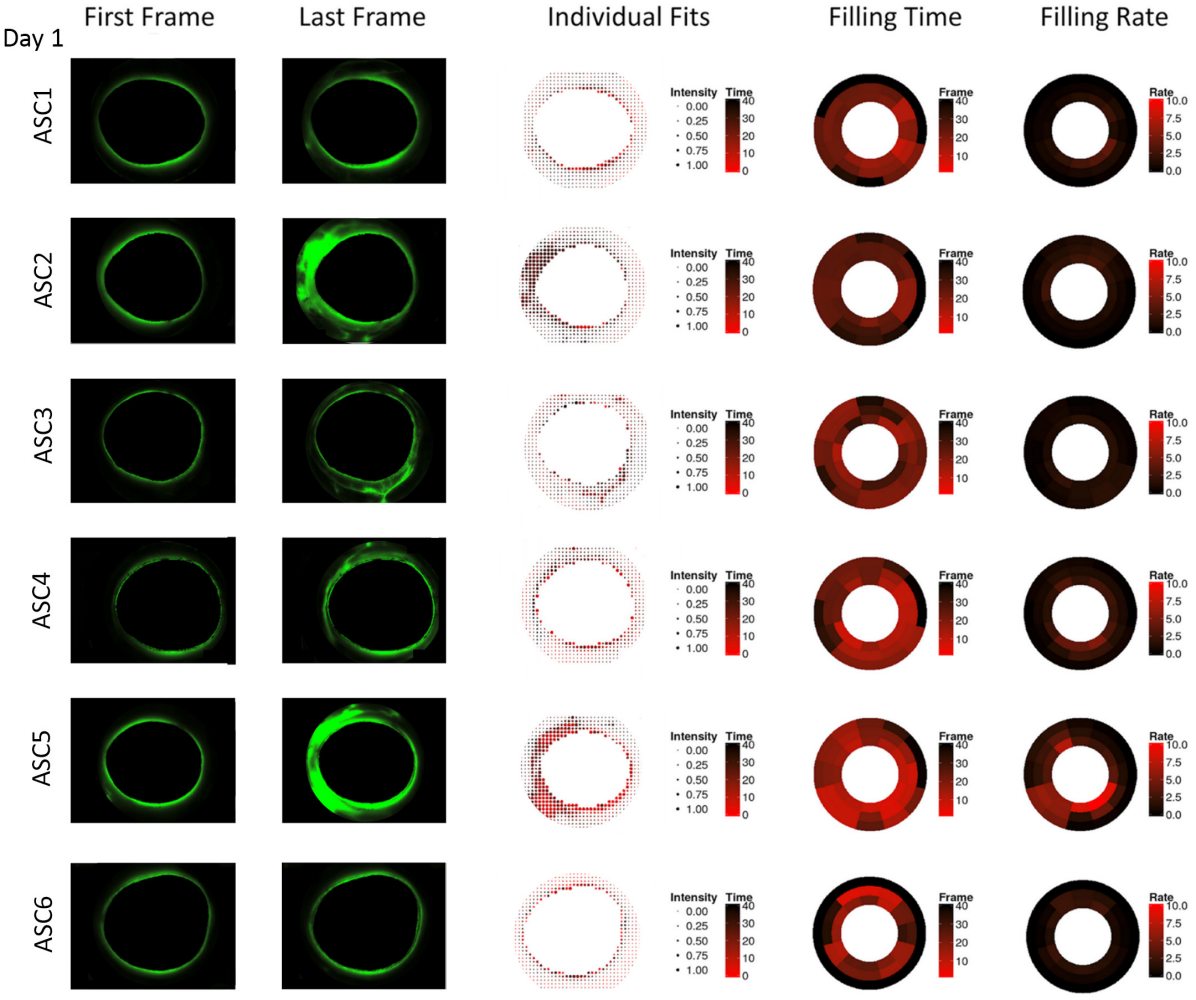
Anterior segment culture canalograms on day 1 of culture with reduced flow. Shown are images of the first and final image analyzed (left), quantitative canalography dot plots using individual fits (center), and ring plots illustrating perilimbal aqueous filling time and filling rate (right). ASC = anterior segment cultures, numbered consecutively. All images are rotated to fit the quadrants pattern used in the other figures (SN, IN, IT, ST in clockwise order).

**Fig 7:**
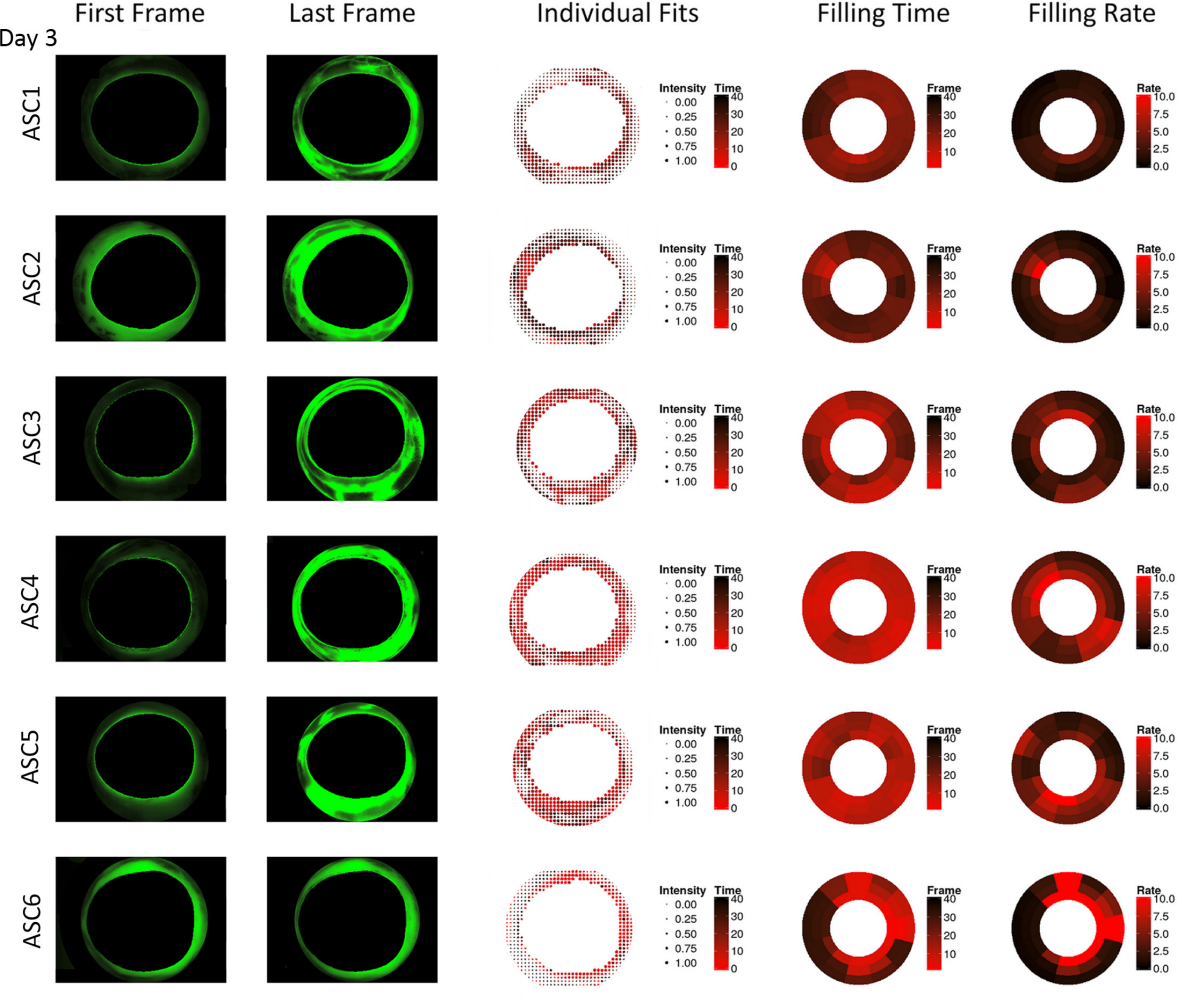
Anterior chamber canalograms on day 3 of culture with improved flow compared to day 1. Shown are images of the first and final image analyzed (left), quantitative canalography dot plots using individual fits (center), and ring plots illustrating perilimbal aqueous filling time and filling rate (right). ASC = anterior segment cultures, numbered consecutively. All images are rotated to fit the quadrants pattern used in the other figures (SN, IN, IT, ST in clockwise order).

In contrast to whole eyes, ASC cultures had a more focal outflow pattern with fewer quadrants contributing to outflow on day 1 but convergence was detected in active quadrants (Fig 6) here as well. On day 3, quantitative canalograms indicated a similar pattern but with faster filling (Fig 7).

## Discussion

Conventional outflow of aqueous humor can be measured by fluorophotometry that analyzes the decreasing fluorescence of fluorescein in the anterior chamber.[25] Using the same chromophore, uveoscleral outflow has been previously assessed by determining the fluorescence of supernatant obtained from homogenized uvea, retina and extraocular tissues.[26] Fluorescein has also been used to visualize the perilimbal microvasculature in rats[27] and to compare outflow patterns in patients after glaucoma surgery.[6,28] In this study, we measured the increase of fluorescence of the same chromophore in the conventional outflow tract with spatial resolution that is higher than in those studies and contains timelapse information. The algorithm eye-canalogram captured images digitally and allowed to describe circumferential as well as regional outflow. The GAM used here combines properties of generalized linear models with additive models by allowing nonlinear relationships between independent and dependent variables.[29] This allows to uncover hidden patterns by using more flexible predictor functions, avoid overfitting due to regularization of predictor functions and are easy to interpret.[30] We have made this code available at GitHub.com.

The outflow tract of pig eyes has been described as the closest match to that of primate eyes[31] with a relatively large, wedge-shaped TM and circumferential drainage elements that are homologous to discontinuous Schlemm’s canal segments. These have been referred to as the “angular aqueous plexus”[32] or “porcine Schlemm’s canal”.[33] In keeping with the recently introduced term “*canalogram”* for imaging outflow in human eyes,[14,34,35] we are using this term here as well.

These experiments confirm the hypothesis that a commonly used glaucoma research model[19,36,37] has an altered outflow pattern compared to whole eyes. Anterior chamber cultures had a more focal filling pattern with poor flow in some quadrants. The faster nasal filling of whole eyes was not seen in anterior chamber cultures. This is likely caused by compression of episcleral veins that are downstream of the collector channels. Increased episcleral venous pressure from ligature or sclerosis can be used to induce elevated intraocular pressure in animal models of glaucoma[38,39] and this might be a tissue culture equivalent with a mild outflow obstruction that is temporary. Vascular endothelia downstream of the TM may develop increased permeability after day 3 of culture further reducing post-TM resistance.

Whole eyes had faster filling nasally, consistent with the presence of larger SC segments and larger aqueous veins compared to the temporal circumference.[40] It matches the location commonly used to enhance outflow in minimally invasive glaucoma surgery.[14] After three days, the poor flow in some quadrants of anterior chamber cultures normalized thereafter suggesting that locally reduced perfusion in ASC segments is eventually overcome by constant perfusion.

The *eye-canalogram* algorithm also measured limbus parallel flow. This outflow component had a pattern that matched the location of larger, radial aqueous veins seen in the canalograms and individual fit graphs.

Pig eyes rendered the advantage of an anatomy and size that is similar to primates eyes[31] while being more readily available, inexpensive and of consistent high quality. The limited circumferential flow observed in our perilimbal analysis that pools into radial aqueous veins may be the functional result of Schlemm’s canal segments that are in close proximity and partially connected.

In conclusion, canalograms using fluorescein were obtained with standard visualization equipment. We developed an automated image analysis algorithm to describe regionally different outflow patterns and filling times. These techniques might be clinically applicable to evaluate the mechanism of local flow enhancement or its failure in minimally invasive glaucoma surgery as well as of cell and gene based therapies of the outflow tract.

## Supporting Information

S1 Fig Eye canalogram image analysis with generalized additive models. (PDF)
