# Supplementary material for: Regionally Discrete Aqueous Humor Outflow Quantification Using Fluorescein Canalograms
Source: PLoS One. 2016 Mar 21;11(3):e0151754. doi: 10.1371/journal.pone.0151754 (PMC4801333; doi:10.1371/journal.pone.0151754)

Eye Canalogram Image Analysis with Generalized Additive Models

Eric N. Brown

2015-10-12

Introduction

This R Markdown document illustrates how to use the `eye-canalogram` package to quantitatively analyze canalograms obtained in aqueous-outflow eye models. R code is provided along with its output.

Load R Packages

The fist step is to load the required `R` packages and others used by this document:

```
library(EyeCanalogram)

library(knitr)
library(reshape2)
library(ggplot2)
library(dplyr)
library(mgcv)
```

Eye Canalogram Image Processsing

Options

Option	Value
Title	AC2 on Day 1
Path	ACs/d1/___13_/_13_T00
Resolution	32
Radius Bands	3

Loading Images

The `read.images` function of the `EyeCanalogram` package is first used to read all canalogram images. The most important function parameter is the `root` parameter which is the common filename prefix for all images. For example, if `root` is `ACs/d1/__13_/_13_T00`, then all TIFF files beginning with `_13_T00` in the `ACs/d1/__13_` directory would be loaded. The `n` parameter can be used to restrict loading to the first `n` images found. The `low` parameter sets the number of macropixels used in each direction. Here `low` is 32 which will result in resizing all images to have 32 pixels in the smallest dimension (here the image height) with possibly more in the other dimension depending on the input image aspect ratio.

Two special files are potentially loaded along with the canalograms. If a file named `mask.tif` or `mask.png` exists, it is converted to a black-and-white mask where white pixels indicate the location and shape of the

cornea in all other canalogram images. If a file `roi.tif` exists, it too is converted to a black-and-white mask where white pixels indicate the perilimbal area and cornea to analyze — black pixels can be used to mask out regions not of interest such as the compression ring used for anterior chamber cultures.

```
images <- read.images(root = root, n = NA, low = resolution)
```

```
## Reading '_13_T00' images in directory '/home/enb/LAB/MANUSCRIPTS/eye-canalogram/test/data-paper-1/ACs
## Using cornea mask found in '/home/enb/LAB/MANUSCRIPTS/eye-canalogram/test/data-paper-1/ACs/d1/__13_/r
## Cornea provided by mask.
## Using region-of-interest mask found in '/home/enb/LAB/MANUSCRIPTS/eye-canalogram/test/data-paper-1/AC
```

This resulted in 41 images being loaded from the /home/enb/LAB/MANUSCRIPTS/eye-canalogram/test/data-paper-1/ACs/d1/___13_ directory. After reducing their size to 43 by 32 pixels (also called macropixels), these images take up 4707256 bytes of memory. These data are stored at `images$data.low`, which is an object of `Image` class from the `EBImage` BioConductor package.

For illustration, next the macropixels are extracted for a number of frames and displayed using `ggplot2`. The `melt` function from the `reshape2` package is used to convert the $43 \times 32 \times 41$ array of pixel information into a `data.frame`. The column names are then assigned, individual frames chosen, and finally those frames are displayed.

```
x <- melt(images$data.low)
colnames(x) <- c('x', 'y', 't', 'I')
frames <- seq(min(x$t), max(x$t), by = 5)
ggplot(x[x$t %in% frames,], aes(x, y, fill = I)) +
  coord_fixed(ratio = 1) +
  ggtitle("Macropixel Data") + xlab("X") + ylab("Y") +
  geom_raster() + facet_wrap(~ t) +
  scale_fill_gradient("Raw Intensity", low = '#000000', high = '#00FF00') + theme_bw()
```

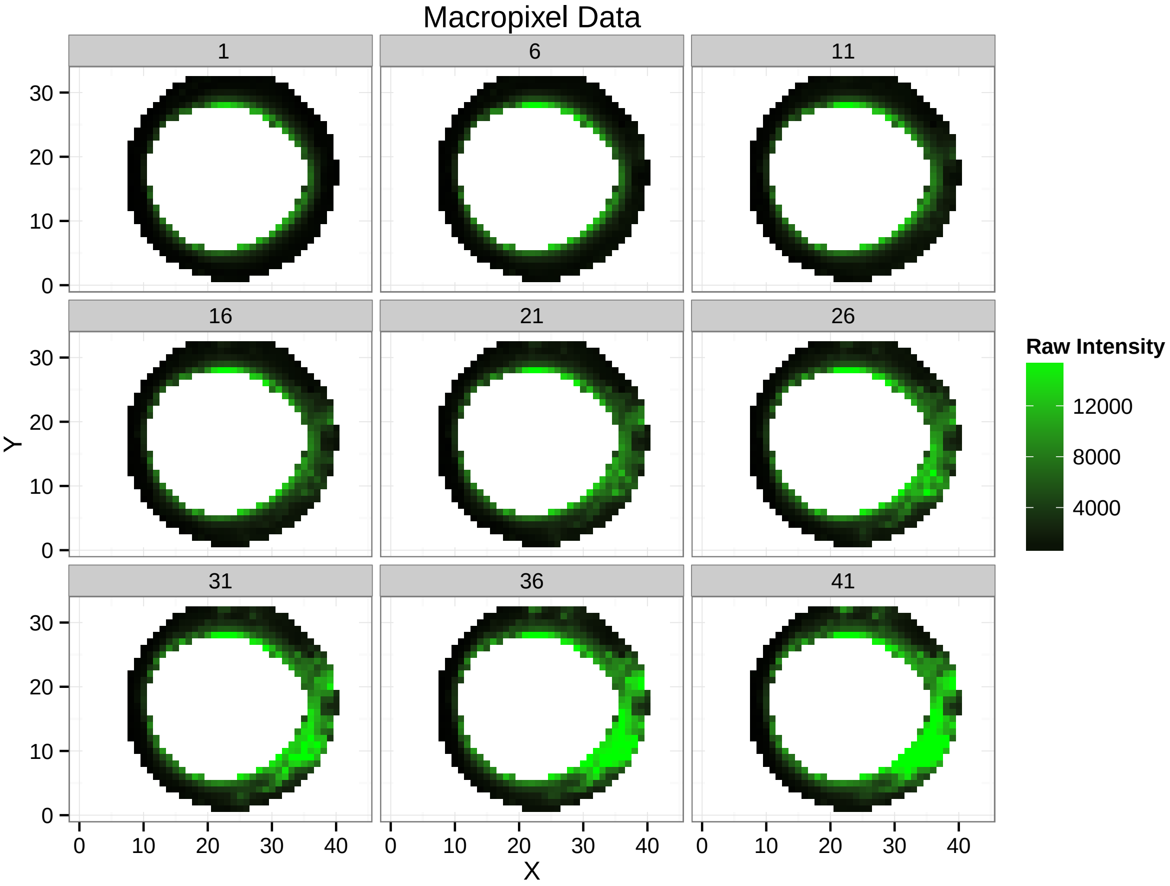

Process Images

The entire dataset is fit individually at each macropixel and all at once (global fit) with smoothing along the clock hours, radial distance, time, and a tensor product for the interactions. The `fit.gam` function of the `EyeCanalogram` package does all of the work. The function parameter `r_bands` sets how many radial bands around the cornea are used for the global fit and ring plots.

```
processed <- fit.gam(images, r_bands = r_bands)
```

```
## Fitting GAM to grid (individual fits)
##   Fitting 421 individual GAM models
## Processing grid metrics
```

```
## Fitting GAM to rings (global fit)
## Fitting low-resolution data
## Processing global & ring metrics
## Processing quadrants
## Updating rings
```

Quadrant Flow

Flow below is estimated from the increase in fluorescence (from the initial, possibly non-zero, fluorescence in the first frame) to the time at half-max. [This differs from the *Filling Rate* which is the is the increase in absolute fluorescence (from an initial value of zero) to the time at half-max.] The flow is determined for a thin region a given distance from the cornea. Here, a distance of 2 pixels from the cornea is chosen. The global-fit is used to estimate the fluorescence in this region and then flow is calculated. The proportion of the total flow for the entire analysis region (outside of the cornea and within the mounting ring) is then attributed to each quadrant. This can be converted into an estimate in microliters/minute given an estimated 3 microliters of total flow flow per minute for the entire eye.

```
# Compute the flow per quadrant
flow <- processed$gam$prediction_metrics %>%
  dplyr::group_by(quadrant) %>%
  dplyr::summarize(min_theta = floor(min(theta %% 360)),
                   max_theta = ceiling(max(theta %% 360)),
                   mean_rate = mean(mid_rate),
                   mean_flow = mean(flow),
                   total_flow = sum(flow))
flow$percent_flow <- 100 * flow$total_flow / sum(flow$total_flow)
# Give the quadrants nice names
flow$quadrant <- '??'
flow$quadrant[flow$min_theta < 45 & 45 < flow$max_theta] <- 'SN'
flow$quadrant[flow$min_theta < 135 & 135 < flow$max_theta] <- 'IN'
flow$quadrant[flow$min_theta < 225 & 225 < flow$max_theta] <- 'IT'
flow$quadrant[flow$min_theta < 315 & 315 < flow$max_theta] <- 'ST'
# Pull out only the info for the table
flow <- flow %>%
  select(quadrant, total_flow, percent_flow) %>%
  mutate(flow = percent_flow / 100 * 3.0)
# Print the table
kable(flow)
```

quadrant	total_flow	percent_flow	flow
SN	1.069871	15.55509	0.4666527
IN	2.850337	41.44167	1.2432501
IT	1.085844	15.78733	0.4736198
ST	1.871896	27.21591	0.8164774

In the above code, the `processed$gam$prediction_metrics` dataframe contains the predicted fluorescence intensity from the global prediction for a range of all angles, radii, and timepoints. These are then grouped by quadrant (using the `dplyr` package) and the total flow and percent flow is calculated. The next lines assign user-friendly labels to the the `quadrant` column and output it using the `kable` function in the `knitr` package.

Displaying Results

All of the data can be displayed at once as shown above. Alternatively, different measures of the fit can be displayed schematically.

```
# Plot the dot-plot for this eye
print(plot.dot(processed, scale.range = c(1, 3), time.max = 40))
```

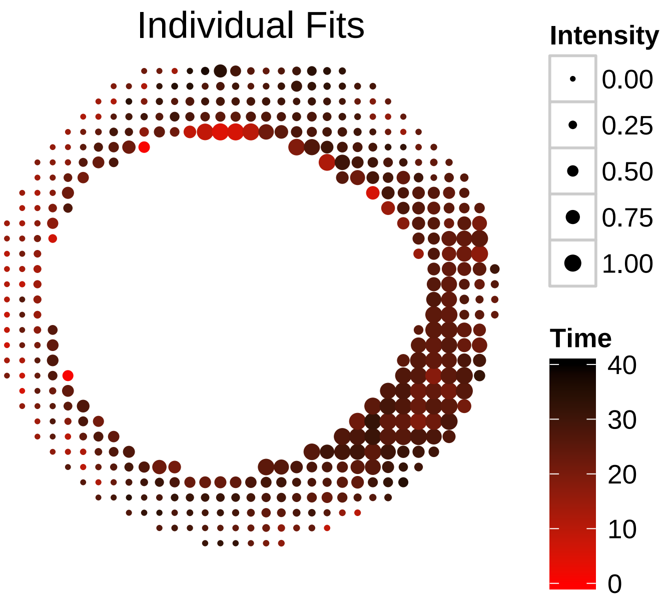


```
# Plot the ring-plot for half-intensity
print(plot.ring(processed, which = 'intensity', resolution = 400))
```

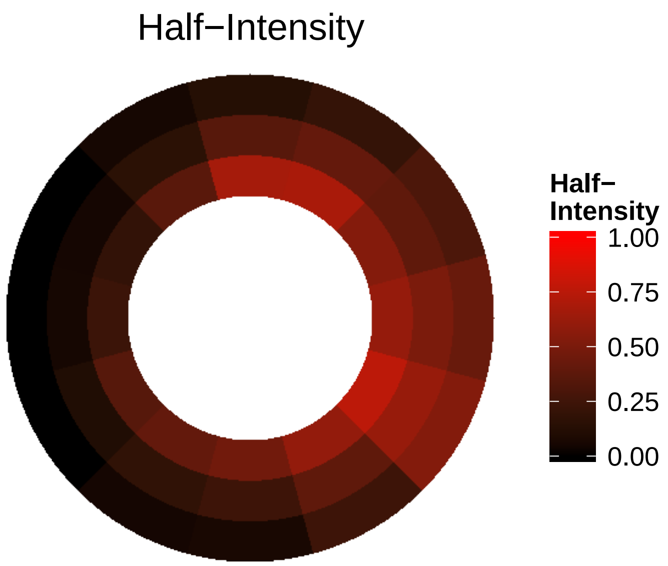


```
# Plot the ring-plot for filling half-time
print(plot.ring(processed, which = 'time', resolution = 400))
```

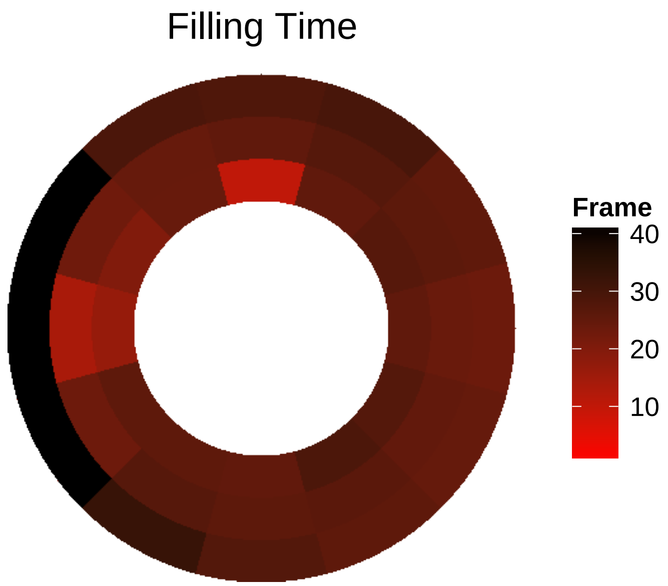

```
# Plot the ring-plot for filling rate
print(plot.ring(processed, which = 'rate', resolution = 400))
```

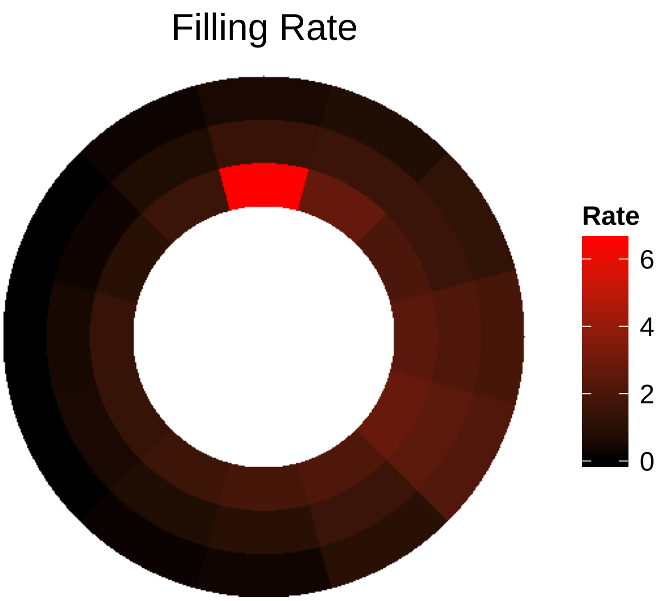

Since all plots are created using the `ggplot2` package, they can be manipulated using the `ggplot2` and `grid` packages:

```
mytheme <- ggplot2::theme_bw() +
  ggplot2::theme(legend.position="right",
                 axis.ticks = ggplot2::element_blank(),
                 axis.text.x = ggplot2::element_blank(),
                 axis.text.y = ggplot2::element_blank(),
                 panel.border = ggplot2::element_blank(),
                 panel.grid = ggplot2::element_blank(),
                 panel.margin = grid::unit(0, 'mm'),
                 legend.margin = grid::unit(0, 'mm'),
                 legend.key.size = grid::unit(10, "mm"),
                 legend.key = ggplot2::element_rect(fill = NULL, color = "white"),
                 legend.box = 'horizontal',
                 plot.margin = grid::unit(c(0,0,0,0), 'mm'))

p1 <- plot.dot(processed, scale.range = c(1, 2), time.max = 40) +
  mytheme + ylab("") + ggtitle("")
p2 <- plot.ring(processed, which = 'time', resolution = 400, time.max = 40) +
  mytheme + ggtitle("")
p3 <- plot.ring(processed, which = 'rate', resolution = 400, rate.max = 10) +
  mytheme + ggtitle("")

grid::grid.newpage()
grid::pushViewport(grid::viewport(layout = grid::grid.layout(nrow = 1, ncol = 3, widths = grid::unit(c(
```

```r
print(p1, vp = grid::viewport(layout.pos.row = 1, layout.pos.col = 1))
print(p2, vp = grid::viewport(layout.pos.row = 1, layout.pos.col = 2))
print(p3, vp = grid::viewport(layout.pos.row = 1, layout.pos.col = 3))
```

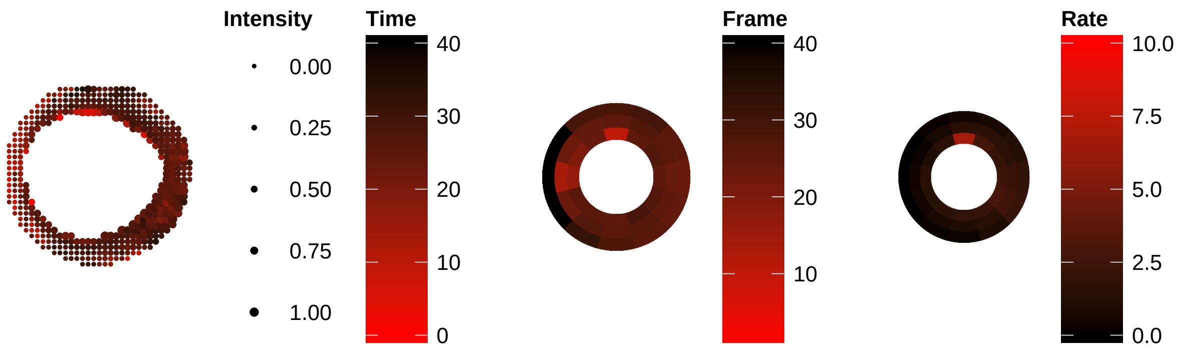

Supplement: S1 Fig — (PDF) [file pone.0151754.s001.pdf]
